# Natural history of malignant bone disease in breast cancer and the use of cumulative mean functions to measure skeletal morbidity

**DOI:** 10.1186/1471-2407-9-272

**Published:** 2009-08-06

**Authors:** Pierre P Major, Richard J Cook, Allan Lipton, Matthew R Smith, Evangelos Terpos, Robert E Coleman

**Affiliations:** 1 McMaster University, Juravinski Cancer Centre, Hamilton, Ontario, Canada; 2 University of Waterloo, Waterloo, Ontario, Canada; 3 Penn State Cancer Center, Milton S. Hershey Medical Center, Hershey, Pennsylvania, USA; 4 Massachusetts General Hospital, Boston, Massachusetts, USA; 5 251 General Airforce Hospital, Athens, Greece; 6 Cancer Research Centre, Weston Park Hospital, Academic Unit of Oncology, Sheffield, UK

## Abstract

**Background:**

Bone metastases are a common cause of skeletal morbidity in patients with advanced cancer. The pattern of skeletal morbidity is complex, and the number of skeletal complications is influenced by the duration of survival. Because many patients with cancer die before trial completion, there is a need for survival-adjusted methods to accurately assess the effects of treatment on skeletal morbidity.

**Methods:**

Recently, a survival-adjusted cumulative mean function model has been generated that can provide an intuitive graphic representation of skeletal morbidity throughout a study. This model was applied to the placebo-control arm of a pamidronate study in patients with malignant bone disease from breast cancer.

**Results:**

Analysis by bone lesion location showed that spinal metastases were associated with the highest cumulative mean incidence of skeletal-related events (SREs), followed by chest and pelvic metastases. Metastases located in the extremities were associated with an intermediate incidence of SREs, and those in the skull were associated with the lowest incidence of SREs.

**Conclusion:**

Application of this model to data from the placebo arm of this trial revealed important insight into the natural history of skeletal morbidity in patients with bone metastases. Based on these observations, treatment for the prevention of SREs is warranted regardless of lesion location except for metastases on the skull.

## Background

Malignant bone disease involves a complex interplay between tumor and bone, resulting in increased bone resorption, stimulation of tumor growth in bone, and decreased skeletal integrity[1] The development of bone lesions is common in many types of advanced cancer, including multiple myeloma and breast, prostate, and lung cancers[2] For example, approximately 65% to 75% of patients who develop metastatic disease from breast or prostate cancer (the most common malignancies in women and men, respectively) will develop bone metastases[2,3] Bone metastases from breast cancer have an especially high propensity to cause skeletal complications [4-7] Therefore, a large population of patients are at risk for skeletal-related events (SREs) such as pathologic fractures, spinal cord compression, severe bone pain requiring palliative radiotherapy, loss of structural integrity and impending fracture that requires surgery to bone, and hypercalcemia of malignancy[1] Patients with advanced breast cancer have a median survival of approximately 2 years after an initial diagnosis of bone metastases and are at long-term risk for SREs. These patients experience an average of 3 to 4 SREs every year in the absence of bisphosphonate therapy[1] However, the risk of experiencing subsequent SREs increases approximately 2-fold after the first incident; therefore, SREs usually occur in clusters and become more frequent as the disease progresses[3,8] Skeletal morbidity also undermines patients' quality of life and their ability to function in daily living, and fractures have been associated with significantly reduced survival[9,10]

Currently, data on prognostic indicators for disease progression are available in patients with metastatic bone disease. For example, outcomes from exploratory analyses indicate that the risk of skeletal complications in patients with breast or prostate cancer is correlated with the rate of bone resorption[11,12] Elevated baseline levels of N-terminal telopeptide of type I collagen (NTX), a marker of bone resorption, strongly correlated with the number of SREs or death compared with low NTX levels. Patients with NTX levels > 100 nmol/mmol creatinine were more likely to experience an SRE and/or death than were patients with NTX levels below this level (*p *≤ 0.01). In addition, the number of bone lesions has also been found to influence the rate of skeletal complications from metastatic bone disease. In exploratory analyses of 3 multicenter, phase III trials in patients with metastatic bone disease (n = 1,616) who were retrospectively stratified by the number of bone lesions at baseline, patients with > 3 bone lesions had a higher skeletal morbidity rate than did those with ≤ 3 bone lesions[13] Finally, the presence or absence of pain at baseline may provide insight into a patient's risk for SREs. Retrospective data from a phase III trial suggest that patients with breast cancer and pain at baseline have a higher mean annual incidence of SREs than do patients with no pain at baseline[14]

Insight into the natural course of metastatic bone disease could be enhanced by robust analyses for determining the risk for SREs based on lesion sites. However, such analyses are lacking. The placebo arm of a phase III pamidronate registration study provides an opportunity to investigate the natural history of skeletal morbidity in patients with breast cancer[15] This trial is the largest database of patients with bone metastases from breast cancer who received placebo. The data from this study can serve to illustrate the complicated patterns of skeletal morbidity in patients with advanced breast cancer and provide a rich database for testing the newer statistical methodology for assessing skeletal morbidity, such as random-effects models. This methodology can accommodate variations in event rates between patients, in contrast with previous methodologies, which underestimated data variability and could thereby inflate false-positive errors in treatment comparisons[16] The analyses presented herein assessed the potential prognostic significance of multiple variables including age, location of metastases, and pain to determine survival and SREs. In addition, the relationship of lesion site with risk for SREs was assessed to provide insight on the risk for SREs in patients with advanced cancer.

## Methods

### Study Design

An exploratory analysis was performed using data from the placebo-control arm of a 2-year, randomized, controlled trial of pamidronate for the prevention of skeletal morbidity in patients with bone metastases from breast cancer who were receiving cytotoxic chemotherapy. Patients were enrolled from January 1991 through March 1994, and trial results were published in December 1996[15] The rationale for the trial size was based on SRE prevention by pamidronate versus placebo and has been previously described[15] Briefly, patients with stage IV breast cancer who had at least 1 osteolytic bone metastasis were stratified by Eastern Cooperative Oncology Group (ECOG) performance status and then randomized to receive either pamidronate 90 mg (via 2-hour infusion) or placebo every 3 to 4 weeks for 1 year. Patients who completed 1 year on study were allowed to continue on study for an additional 1 year of therapy. The primary endpoint was the time to first SRE, including pathologic fracture, spinal cord compression, and the requirement for surgery or radiation therapy to bone. Secondary endpoints included incidence of SREs, change in bone pain and performance status, and overall survival. All SREs were included in the current analysis, in contrast with the original study report in which a 21-day window between on-study SREs was used. Lesion sites were assigned for bone metastases on the basis of anatomic criteria: the pelvis included any non-spinal lesion visible on a standard pelvic radiograph (including the approximate top one third of the femur), the skull included any lesion on the skull including the jaw, the chest included any lesions on the chest, rib, or collar bone (excluding the spine), and the extremities included any lesion in a region not described by the other sites (eg, the arms, lower legs, hands, and feet). The locations of SRE sites were determined by review of medical records, which was necessary because of the presence of multiple bone lesion sites in most patients.

### Statistical Methodology

Associations between sites of bone lesions were assessed using descriptive statistics. Cox proportional hazard models were used for univariate and multivariate analyses of potential risk factors for reduced survival, time to first SRE, and time to first pathologic fracture. All covariates were included in a multivariate model, and then only variables retaining significance (*p *< 0.05) were included in a reduced multivariate model. Baseline variables considered included age (< 50 or ≥ 50 years old), time from diagnosis of cancer to study entry (years), time from diagnosis of metastases to study entry (years), urinary hydroxyproline/creatinine ratio, serum bone-specific alkaline phosphatase (BALP) level (in units/L), Brief Pain Inventory pain score, fracture history (yes/no), radiotherapy history (yes/no), chemotherapy history (yes/no), hormonal therapy history (< 2 or ≥ 2), ECOG performance status (< 2 or 2 to 3), estrogen- and progesterone-receptor status (negative, positive, or unknown), metastatic disease limited to skeleton (yes/no), presence of lung or liver metastases (yes/no), presence and quantity of primarily osteolytic, osteoblastic, or mixed bone lesions (0, 1 to 2, or ≥ 3), and lesion site. The survival distribution was assessed and stratified by bone lesion site using Kaplan-Meier estimates. For each bone lesion site, skeletal morbidity parameters for that site were assessed using the survival-adjusted cumulative mean function[17,18] Site-specific SREs by each respective lesion site were evaluated in a survival-adjusted model. This model has previously been used to capture patterns of skeletal morbidity during bone-targeted therapy[16,19]

All SREs at each respective site were included in the cumulative mean functions, and no event window was applied to the SREs because all SREs contribute to the overall burden of skeletal morbidity, even if they are related events. Although hypercalcemia of malignancy is a clinically important SRE associated with bone metastases, the anatomic site responsible for its cause cannot be specified; therefore, it was not included in the survival-adjusted cumulative mean functions for each lesion site.

## Results

### Patient Demographics and Baseline Characteristics

Bone lesion data were available for all 195 patients in the placebo group. Patient demographic and baseline disease characteristics are shown in Table 1[15] The skeleton was the only site of metastasis in 60% of patients. Consistent with the fact that no bisphosphonates had yet been approved in this setting at the time, most patients had been diagnosed with bone metastases more than 1 year before study entry, but none had received prior bisphosphonate therapy. Most patients also had bone lesions at multiple anatomic locations. The majority of patients (64%) were found to have bone metastases in the pelvis, spine, and chest (Table 2).

**Table 1:** Patient demographics and baseline disease characteristics

Characteristics	Placebo group (n = 195)
Age, mean years ± SD	56 ± 12
Patients < 50 years of age, n (%)	67 (34)
ECOG performance status, n (%)	
0 – 1	128 (66)
2 – 3	67 (34)
Estrogen- and progesterone-receptor status, n (%)	
Positive for at least 1	120 (62)
Other	75 (38)
Sites of metastasis, patients, n (%)	
Bone, any	195 (100)
Bone as only metastatic site	117 (60)
Lung	30 (15)
Liver	29 (15)
Brain	1 (1)
Other	27 (14)
Time from primary to bone metastases diagnosis, median years ± SD	3.8 ± 4.5
Time from bone metastases to study entry, mean years ± SD	1.6 ± 1.7
Patients with bone lesions ≥ 1 cm in diameter, n (%)	
1 lesion	82 (42)
2 lesions	71 (36)
≥ 3 lesions	42 (22)
Lesion types and numbers	
1 – 2 osteolytic	104 (53)
≥ 3 osteolytic	91 (47)
0 osteoblastic	146 (75)
1 – 2 osteoblastic	37 (19)
≥ 3 osteoblastic	12 (6)
0 mixed lesions	28 (14)
1 – 2 mixed lesions ≥ 3 mixed lesions	61 (31) 106 (54)
Patients with SREs during the 3 months before study entry, n (%)	
Palliative radiotherapy	57 (29)
Fracture	35 (18)
Pain scores, n (%)	
0	27 (14)
1 – 3	76 (39)
4 – 9	92 (47)
Prior therapy regimens, n (%)	
0 – 1 chemotherapy	80 (41)
2 – 3 chemotherapy	104 (53)
≥ 4 chemotherapy	11 (6)
0 – 1 hormonal therapy	102 (52)
2 – 3 hormonal therapy	77 (39)
≥ 4 hormonal therapy	16 (8)

SD = Standard deviation, ECOG = Eastern Cooperative Oncology Group, SRE = Skeletal-related event.

Adapted with permission from Hortobagyi et al. Efficacy of pamidronate in reducing skeletal complications in patients with breast cancer and lytic bone metastases. Protocol 19 Aredia Breast Cancer Study Group. *N Engl J Med*. 1996;335:1785–1791. Copyright^© ^1996 Massachusetts Medical Society. All rights reserved[15]

**Table 2:** Number of patients with at least 1 baseline lesion at each indicated location

		Patients with additional lesions at indicated locations, n (%)					
		
Lesion location	Patients with ≥ 1 lesion at this location, n	Pelvis	Chest	Spine	Skull	Other	Only
Pelvis	178	--	131 (74)	158 (89)	118 (66)	36 (20)	3 (2)
Chest	142	131 (92)	--	122 (86)	101 (71)	27 (19)	3 (2)
Spine	161	158 (98)	122 (76)	--	111 (69)	34 (21)	0
Skull	132	118 (89)	101 (77)	111 (84)	--	27 (21)	0
Other	42	36 (86)	27 (64)	34 (81)	27 (64)	--	0

### Risk Factors for Skeletal Morbidity and Death

For overall survival, all of the variables assessed in both univariate and full multivariate analyses and their associated relative risk ratios are shown in Tables 3 and 4. At trial completion, 43% of patients were alive. Hydroxyproline/creatinine ratio (centered) and positive progesterone-receptor status were the only variables to significantly correlate with overall survival in both univariate (*p *= 0.001 and *p *= 0.022, respectively) and full multivariate analyses (*p *= 0.001 and *p *= 0.050, respectively). The same variables used for overall survival were included in the univariate and full multivariate analyses to assess prognostic factors for experiencing a first SRE (Tables 5 and 6). At trial completion, 56% of patients had experienced at least 1 SRE. Pain scores and prior radiotherapy significantly correlated with risk of first SRE in both univariate (*p *< 0.001 for all) and full multivariate analyses (*p *< 0.001 for pain; *p *= 0.018 for prior radiotherapy).

**Table 3:** Univariate analysis for overall survival

Variable	RR	(95% CI)	*p *value
Age ≥ 50 years	0.96	(0.68, 1.35)	0.802
**ECOG performance status**			
**2 – 3 versus 0 – 1**	**1.49**	**(1.06, 2.12)**	**0.024**
**Hydroxyproline/creatinine ratio (centered)**	**1.04**	**(1.02, 1.07)**	**0.001**
Bone-specific alkaline phosphatase (U/L)	1.00	(1.00, 1.01)	0.415
Estrogen-receptor status versus negative			
Positive	0.67	(0.42, 1.06)	0.085
Unknown	0.96	(0.64, 1.44)	0.857
**Progesterone-receptor status versus negative**			
**Positive**	**0.61**	**(0.40, 0.93)**	**0.022**
**Unknown**	**0.70**	**(0.49, 0.99)**	**0.047**
Sites of metastasis (yes versus no)			
Bone as only metastatic site	0.90	(0.65, 1.26)	0.553
Lung	1.43	(0.89, 2.30)	0.139
Liver	1.12	(0.69, 1.79)	0.653
Time from diagnosis of bone metastases to study entry, years	1.10	(1.00, 1.21)	0.059
Time from cancer diagnosis to study entry, years Lesion characteristics and numbers	1.00	(0.95, 1.03)	0.630
≥ 3 osteolytic versus < 3 osteolytic	1.29	(0.93, 1.80)	0.128
1 – 2 osteoblastic versus no osteoblastic	0.98	(0.65, 1.48)	0.935
≥ **3 osteoblastic versus no osteoblastic**	**0.75**	**(0.62, 0.91)**	**0.003**
1 – 2 mixed versus no mixed	0.81	(0.48, 1.36)	0.423
≥ 3 mixed versus no mixed	1.03	(0.67, 1.58)	0.896
Prior fracture (yes versus no)	1.18	(0.78, 1.77)	0.440
**Pain scores (centered)**	**1.07**	**(1.01, 1.13)**	**0.034**
Prior chemotherapy (yes versus no)	1.85	(0.59, 5.87)	0.294
≥ 2 prior hormonal therapies (yes versus no)	1.31	(0.94, 1.82)	0.110
Prior radiotherapy (yes versus no)	1.15	(0.80, 1.64)	0.464

Values in bold represent statistically significant correlations.

RR = Relative risk, CI = Confidence interval, ECOG = Eastern Cooperative Oncology Group, U = Units.

**Table 4:** Full multivariate analysis for overall survival

Variable	RR	(95% CI)	*p *value
Age ≥ 50 years	1.18	(0.78, 1.78)	0.434
ECOG performance status			
2 – 3 versus 0 – 1	1.15	(0.76, 1.76)	0.507
**Hydroxyproline/creatinine ratio (centered)**	**1.06**	**(1.02, 1.10)**	**0.001**
Bone-specific alkaline phosphatase (U/L)	1.00	(0.99, 1.01)	0.471
Estrogen-receptor status versus negative			
Positive	0.78	(0.44, 1.40)	0.406
Unknown	1.53	(0.67, 3.50)	0.314
**Progesterone-receptor status versus negative**			
**Positive**	**0.58**	**(0.33, 1.00)**	**0.050**
Unknown	0.51	(0.24, 1.07)	0.074
Sites of metastasis (yes versus no)			
Bone as only metastatic site	0.75	(0.44, 1.29)	0.301
Lung	1.49	(0.733, 3.01)	0.273
Liver	0.84	(0.435, 1.61)	0.593
**Time from diagnosis of bone metastases to study entry, years**	**1.21**	**(1.05, 1.38)**	**0.008**
**Time from cancer diagnosis to study entry, years**	**0.83**	**(0.72, 0.95)**	**0.008**
Lesion characteristics and numbers			
≥ 3 osteolytic versus < 3 osteolytic	1.34	(0.92, 1.95)	0.128
1 – 2 osteoblastic versus no osteoblastic	1.19	(0.74, 1.92)	0.471
≥ 3 osteoblastic versus no osteoblastic	0.79	(0.36, 1.74)	0.559
1 – 2 mixed versus no mixed	0.64	(0.36, 1.16)	0.140
≥ 3 mixed versus no mixed	0.63	(0.35, 1.12)	0.112
Prior fracture (yes versus no)	0.99	(0.58, 1.67)	0.964
Pain scores (centered)	1.03	(0.96, 1.11)	0.406
Prior chemotherapy (yes versus no)	1.54	(0.46, 5.16)	0.484
≥ **2 prior hormonal therapies (yes versus no)**	**1.61**	**(1.08, 2.40)**	**0.020**
Prior radiotherapy (yes versus no)	0.75	(0.47, 1.20)	0.230

Values in bold represent statistically significant correlations.

RR = Relative risk, CI = Confidence interval, ECOG = Eastern Cooperative Oncology Group, U = Units.

**Table 5:** Univariate analysis for first skeletal-related event

Variable	RR	(95% CI)	*p *value
Age ≥ 50 years	0.94	(0.63, 1.40)	0.760
**ECOG performance status**			
**2 – 3 versus 0 – 1**	**1.81**	**(1.21, 2.72)**	**0.004**
Hydroxyproline/Creatinine ratio (centered)	1.00	(0.97, 1.04)	0.921
Bone-specific alkaline phosphatase, U/L	1.01	(1.00, 1.01)	0.204
**Estrogen-receptor status versus negative**			
**Positive**	**0.56**	(**0.34, 0.92)**	**0.023**
Unknown	0.89	(0.58, 1.36)	0.580
Progesterone-receptor status versus negative			
Positive	0.62	(0.38, 1.01)	0.053
Unknown	0.78	(0.52, 1.16)	0.214
Sites of metastasis (yes versus no)			
Bone as only metastatic site	0.84	(0.57, 1.24)	0.392
Lung	1.32	(0.77, 2.29)	0.317
Liver	1.26	(0.75, 2.13)	0.379
Time from diagnosis of bone metastases to study entry, years	1.00	(0.95, 1.04)	0.912
Time from cancer diagnosis to study entry, years	1.00	(0.96, 1.04)	0.947
**Lesion characteristics and numbers**			
**≥ 3 osteolytic versus < 3 osteolytic**	**1.92**	**(1.29, 2.84)**	**0.001**
1 – 2 osteoblastic versus no osteoblastic	1.28	(0.82, 2.02)	0.282
**≥ 3 osteoblastic versus no osteoblastic**	**0.39**	**(0.32, 0.50)**	**< 0.001**
1 – 2 mixed versus no mixed	1.16	(0.64, 2.09)	0.624
≥ 3 mixed versus no mixed	1.26	(0.77, 2.06)	0.352
**Prior fracture (yes versus no)**	**1.78**	**(1.12, 2.81)**	**0.014**
**Pain scores (centered)**	**1.29**	**(1.19, 1.40)**	**< 0.001**
Prior chemotherapy (yes versus no)	0.89	(0.32, 2.48)	0.816
≥ 2 prior hormonal therapies (yes versus no)	1.11	(0.75, 1.63)	0.605
**Prior radiotherapy (yes versus no)**	**2.02**	**(1.35, 3.02)**	**< 0.001**

Values in bold represent statistically significant correlations.

RR = Relative risk, CI = Confidence interval, ECOG = Eastern Cooperative Oncology Group, U = Units.

**Table 6:** Full multivariate analysis for first skeletal-related event

Variable	RR	(95% CI)	*p *value
Age ≥ 50 years	1.12	(0.69, 1.83)	0.651
ECOG performance status			
2 – 3 versus 0 – 1	1.33	(0.78, 2.25)	0.296
Hydroxyproline/Creatinine ratio (centered)	0.97	(0.92, 1.03)	0.292
Bone-specific alkaline phosphatase, U/L	1.01	(1.00, 1.02)	0.083
Estrogen-receptor status versus negative			
Positive	0.94	(0.50, 1.76)	0.838
Unknown	0.91	(0.35, 2.36)	0.844
Progesterone-receptor status versus negative			
Positive	0.80	(0.41, 1.55)	0.506
Unknown	1.19	(0.51, 2.81)	0.685
Sites of metastasis (yes versus no)			
Bone as only metastatic site	0.81	(0.45, 1.44)	0.467
Lung	1.49	(0.70, 3.20)	0.306
Liver	0.98	(0.48, 1.99)	0.960
Time from diagnosis of bone metastases to study entry, years	1.01	(0.84, 1.20)	0.992
Time from cancer diagnosis to study entry, years	0.97	(0.91, 1.04)	0.395
**Lesion characteristics and numbers**			
≥ 3 osteolytic versus < 3 osteolytic	1.53	(0.96, 2.44)	0.071
**1 – 2 osteoblastic versus no osteoblastic**	**1.70**	**(1.01, 2.86)**	**0.045**
≥ 3 osteoblastic versus no osteoblastic	0.70	(0.24, 2.08)	0.525
1 – 2 mixed versus no mixed	1.16	(0.57, 2.33)	0.687
≥ 3 mixed versus no mixed	1.47	(0.72, 3.01)	0.291
Prior fracture (yes versus no)	0.83	(0.44, 1.58)	0.577
**Pain scores (centered)**	**1.27**	**(1.15, 1.40)**	**< 0.001**
Prior chemotherapy (yes versus no)	1.07	(0.33, 3.41)	0.915
≥ 2 prior hormonal therapies (yes versus no)	1.00	(0.62, 1.62)	1.000
**Prior radiotherapy (yes versus no)**	**1.84**	**(1.11, 5.03)**	**0.018**

Values in bold represent statistically significant correlations.

RR = Relative risk, CI = Confidence interval, ECOG = Eastern Cooperative Oncology Group, U = Units.

**Figure 1:**
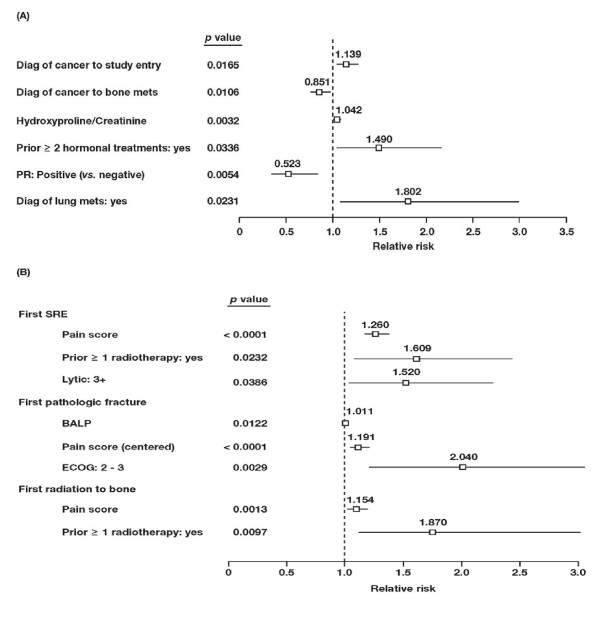
**Forest plot of relative risks in patients with bone metastases from breast cancer**. Significant covariates for (A) overall survival, (B) first SRE, first pathologic fracture, and first radiation to bone. The relative risk for each covariate was derived from reduced multivariate analyses. Lines represent 95% confidence intervals. PR = Progesterone receptor, SRE = Skeletal-related event, BALP = Bone-specific alkaline phosphatase, ECOG = Eastern Cooperative Oncology Group.

The reduced multivariate models included all significant variables from the multivariate models. For overall survival (Figure 1A), increased time from diagnosis of cancer to study entry, a higher hydroxyproline/creatinine ratio, a history of 2 or more prior hormonal therapies, and the presence of lung metastases significantly correlated with decreased survival duration (*p *≤ 0.034) in the reduced model. Increased time from diagnosis of cancer to diagnosis of bone metastases and positive progesterone-receptor status significantly correlated with increased survival (*p *≤ 0.011). For first SRE, a higher pain score, prior radiotherapy, and the presence of 3 or more osteolytic lesions correlated with a significantly increased risk (*p *≤ 0.039; Figure 1B). For pathologic fractures, a higher level of BALP, a higher pain score, and a poorer performance status (ECOG 2 or 3) significantly correlated with increased risk (*p *≤ 0.012; Figure 1B). For radiotherapy to bone, a higher pain score and prior radiotherapy correlated with an increased risk (*p *≤ 0.010; Figure 1B).

### Cumulative Mean Function for Bone Lesion Locations

**Figure 2:**
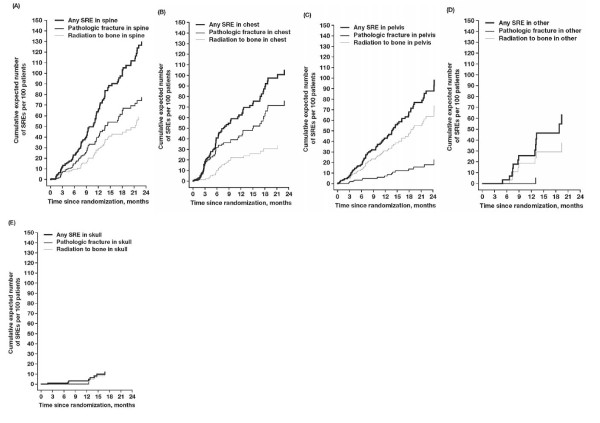
**Time course of cumulative mean events of skeletal-related events (SREs) in patients with bone metastases from breast cancer**. The incidence of SREs was assessed for patients with (A) spinal lesions, (B) thoracic lesions, (C) pelvic lesions, (D) lesions of the extremities, or (E) skull lesions.

Median survival was similar for each of the bone lesion groups and was approximately 15 months. Spinal lesions were associated with the highest incidence of SREs and had a cumulative mean incidence of approximately 0.65 SREs per year (Figure 2A). The most common spinal SREs were palliative radiotherapy and fractures. Thoracic and pelvic lesions were associated with a slightly lower incidence of SREs and had a cumulative mean incidence of approximately 0.50 SREs per year (Figures 2B and 2C, respectively). Lesions located on the extremities were associated with an intermediate incidence of SRE and had a cumulative mean incidence of approximately 0.25 SREs per year (Figure 2D). Lesions located in the skull were associated with the lowest incidence of SREs and had a cumulative mean incidence of < 0.1 SRE per year (Figure 2E). The most common SRE experienced in the skull was the need for radiotherapy.

**Figure 3:**
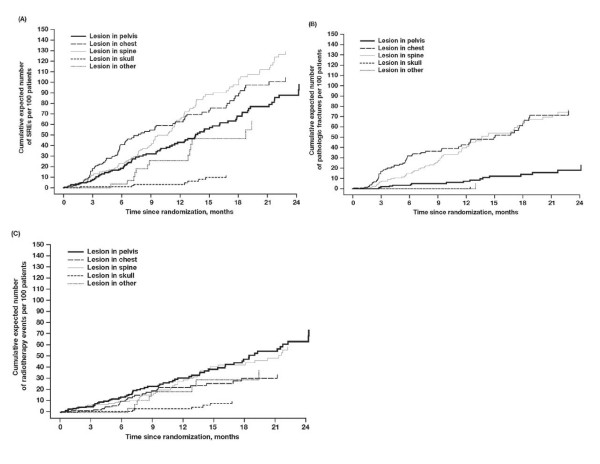
**Time course of survival-adjusted cumulative mean function of skeletal-related events (SREs) in patients with bone metastases from breast cancer**. Lesion locations were determined for patients with (A) any SRE, (B) pathologic fractures, and (C) radiation to bone.

The incidence of SREs for the overall patient population was determined based on SRE location. Spinal lesions carried the highest cumulative mean incidence of SREs, followed by lesions in the chest and pelvis (Figure 3A). Lesions in the chest and spine carried the highest cumulative mean incidence of pathologic fractures at these sites, whereas fractures were less common in the pelvis and rare in the skull or extremities (Figure 3B). Although radiation to bone was the most common SRE for skull lesions, the overall incidence of radiation to bone in the skull was lowest. The incidence of radiation to bone was highest for pelvic and spinal lesions (Figure 3C).

## Discussion

The development of bone metastases is common in many advanced cancers including cancers of the breast, prostate, and lung[9] After diagnosis of bone metastases, the median survival varies among different tumor types but can be measured in months for patients with advanced lung cancer or years for patients with advanced breast or prostate cancer. These patients are at long-term risk for developing painful and potentially debilitating SREs that can negatively influence their quality of life and decrease their duration of survival. Patients with metastatic bone disease will generally experience 3 to 4 SREs each year,[1] although the occurrence of these skeletal complications is not regular. Skeletal-related events are known to occur more frequently during periods of disease progression and occur more often in temporal clusters as the cancer becomes more advanced. Obtaining a greater understanding of the natural course of disease progression in patients with metastatic bone disease may help identify patients who are at higher risk for SREs and may benefit the most from bone-directed therapies such as bisphosphonates, or who may require close monitoring and surveillance.

Information regarding prognostic indicators for disease progression is currently available for patients with metastatic bone disease. For example, variables including prior SRE, number of lesion sites, the presence of pain, and high levels of bone resorption are known to affect patients' risk for SREs. In the current study, reduced multivariate analyses confirmed that the presence of 3 or more osteolytic lesions correlated with an increased risk for SREs. Moreover, multivariate analyses demonstrated that higher pain scores and prior radiotherapy also correlated with an increased risk for SREs. This exploratory natural history study of bone metastases was limited by the available data and only produced statistical associations between variables. The dataset used in these analyses was from the largest documented placebo-controlled trial of patients with bone metastases from breast cancer. Therefore, the evaluated patients were not a random sample of patients with bone metastases, but the best available patients. Moreover, the multiple comparisons used in this study were not prospectively defined at the time of patient enrollment; therefore, there is inconsistency in the number of patients with available data for each variable. Further prospective studies would be needed to confirm the findings from this study. Such trials are unlikely to be initiated, however, as bisphosphonate treatment of patients with bone metastases is now standard of care and a placebo-controlled trial may be unethical.

Although information on prognostic factors for SREs based on baseline disease characteristics is available, data regarding patients' risk for SREs based on the natural course of malignant bone disease are lacking. The data presented herein demonstrate that, in addition to the identified prognostic factors, the complex pattern of skeletal involvement associated with metastatic bone disease is clinically meaningful in determining the risk for SREs. Specifically, the anatomic site of skeletal lesions can provide insight into the risk of skeletal morbidity at the lesion site. For example, patients with pelvic, spinal, and chest lesions can be considered high risk for SREs. These lesions were associated with the highest cumulative mean incidence of SREs per year, with the spine demonstrating the highest risk of SREs. In contrast, lesions on skeletal sites that are not weight-bearing, including the skull and extremities, are associated with fewer SREs. Although the location of skeletal lesions affected the cumulative mean incidence of SREs, it did not appear to affect survival, because the median overall survival was approximately 15 months for all patients regardless of lesion site. However, this analysis is limited by the presence of multiple metastatic sites in the majority of patients, and, while the effects of each lesion site on SRE risk could be evaluated separately by limiting the assessed SREs to those occurring in that lesion site, this type of adjustment could not be made for the survival outcome.

Patients with metastatic bone disease are at a long-term risk for SREs that can undermine patients' functional independence. Identifying those patients who may be more susceptible to potentially debilitating SREs may help to optimize bisphosphonate therapy and maintain their quality of life throughout the course of disease. This study indicates that patients with lesions to the pelvis, spine, or chest are at increased risk of SREs, and prior studies demonstrated that patients with an SRE are at increased risk of subsequent SREs[20] Bisphosphonates reduce the risk of SREs in patients with bone lesions from solid tumors or multiple myeloma and provide continuous treatment benefits to patients even after the development of an SRE[7,20-23] In patients with bone metastases from breast cancer, zoledronic acid reduced the risk of a second SRE by 31% compared with pamidronate (*p *= 0.045)[23] Moreover, in patients with prostate or lung cancer, zoledronic acid reduced the risk of a second SRE by 40% (*p *= 0.028) and 31% (*p *= 0.0009), respectively, compared with placebo[7,22] The combination of these prior findings and the current results indicates that early and continuous bisphosphonate treatment is necessary to delay time to first SRE and reduce the overall occurrence of SREs. Additionally, these analyses underscore the importance of treating bone lesions, especially for patients with pelvis, spine, or rib lesions, and provide important insight into the natural history of bone disease from advanced cancers. The insights gained from these exploratory analyses and previous studies[11,16,24] provide the basis for developing a predictive nomogram for assessing bone morbidity, which we plan to test using the databases from the phase III zoledronic acid clinical trials.

## Conclusion

Skeletal complications from bone metastases can undermine quality of life and may be life-limiting in patients with advanced cancer. Although metastases can occur throughout the skeleton, some sites may be associated with a higher rate of symptoms. Retrospective analysis of the placebo-controlled arm of a pamidronate study in patients with bone metastases from breast cancer revealed that spinal and chest metastases were associated with the highest risk of SREs. In contrast, metastases located in the skull correlated with the lowest risk of SREs. These results provide insight into the natural history of SREs in patients with bone metastases. Moreover, these findings suggest that patients with bone metastases should be treated with bone-targeted therapies to prevent SREs regardless of lesion location.

## Competing Interests Disclosures

P. P. Major has acted as a medical consultant for Novartis Oncology. R. J. Cook has served as a consultant for Novartis. A. Lipton has served on the speakers bureau for Amgen and Novartis; as a consultant for Amgen, Novartis, Merck, Incyte, Monogram Biosciences, Acceleron, and GTX Inc.; as research support for Novartis and Monogram Biosciences, and has given expert testimony for Novartis. M. R. Smith has served as a consultant for Amgen, Merck, and Novartis Oncology and is supported by an NIH Midcareer Clinical Investigator Award (5K24CA121990-02) and research awards from the Prostate Cancer Foundation. E. Terpos has served on an advisory board for Novartis and has received honoraria for participation in Novartis-sponsored satellite symposia. R. E. Coleman has served as a consultant for Novartis, Amgen, and Pfizer; has been a speaker for Novartis, Roche, Pfizer, AstraZeneca, and Amgen; and has received research funding from Novartis and has given expert testimony on their behalf.

## Authors' Contributions

PPM and RJC were responsible for the design and development of the statistical modeling utilized in this study. PPM examined the records for each patient enrolled in the clinical study and classified the locations of each bone lesion and the sites of each of the skeletal-related events that were reported. RJC performed and verified all of the statistical analyses that were included in this study. AL, MRS, ET, and REC each provided strategic direction and critical input into the interpretation and presentation of the analyses. All authors reviewed at least 2 drafts of the manuscript text and approved the final content of this manuscript.

## Pre-publication history

The pre-publication history for this paper can be accessed here:

http://www.biomedcentral.com/1471-2407/9/272/prepub
